# A Compact Broadband Common-Mode Suppression Filter That Integrates Series-Mushroom into Defected Corrugated Reference Plane Structures

**DOI:** 10.3390/s23135852

**Published:** 2023-06-24

**Authors:** Chung-Ke Yu, Ding-Bing Lin, Hsin-Piao Lin, Aloysius Adya Pramudita, Tjahjo Adiprabowo

**Affiliations:** 1 Department of Electronic Engineering, National Taipei University of Technology, Taipei 10608, Taiwan; 2 Department of Electronic and Computer Engineering, National Taiwan University of Science and Technology, Taipei 10607, Taiwan; 3 School of Electrical Engineering, Telkom University, Bandung 40257, Indonesia

**Keywords:** common-mode filter, differential signal, electromagnetic interference, series-mushroom, signal integrity, wideband suppression

## Abstract

This paper proposes a common-mode noise suppression filter scheme for use in the servers and computer systems of high-speed buses such as SATA Express, HDMI 2.0, USB 3.2, and PCI Express 5.0. The filter uses a novel series-mushroom-defected corrugated reference plane (SMDCRP) structure. The measured results are similar to the full-wave simulation results. In the frequency domain, the measured insertion loss of the SMDCRP structure filter in differential mode (DM) can be kept below −4.838 dB from DC to 32 GHz and can maintain signal integrity characteristics. The common-mode (CM) suppression performance can suppress more than −10 dB from 8.81 GHz to 32.65 GHz. Fractional bandwidth can be increased to 115%, and CM noise can be ameliorated by 55.2%. In the time domain, using eye diagram verification, the filter shows complete differential signal transmission capability and supports a transmission rate of 32 Gb/s for high-speed buses. The SMDCRP structure filter reduces the electromagnetic interference (EMI) problem and meets the quality requirements for the controllers and sensors used in the server and computer systems of high-speed buses.

## 1. Introduction

**Figure 1:**
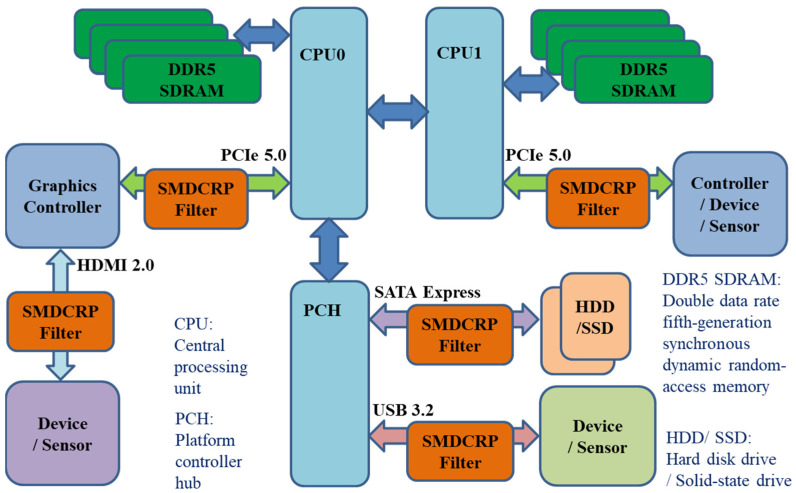
System block diagram of a server with SMDCRP structure filter.

The server and computer products being designed today must continue to have improved processing efficiency and high-speed data transmission. Therefore, the various controllers and sensors in the server need to be connected to the system at higher data transmission speeds. The various buses used within servers need to continuously increase their transmission rates. For instance, the transmission rate of PCI Express 5.0 (PCIe 5.0) needs to be upgraded to 32 Gb/s, that of SATA Express needs to be upgraded to 16 Gb/s, that of USB 3.2 needs to be upgraded to a transmission rate of 10 Gb/s, and that of HDMI 2.0 needs to be upgraded to 18 Gb/s. These are the requirements for current the server design. A system block diagram of a server with a series-mushroom-defected corrugated reference plane (SMDCRP) structure filter is shown in Figure 1. Each controller, device, and sensor are connected to the system using buses, and the SMDCRP structure filter on these buses is used to block related common-mode (CM) noise interference and to improve the stability and accuracy of these controllers, devices, and sensors.

Due to the server’s printed circuit board (PCB) being a compact-sized and high-density component layout, the layout design must consider trace length, thickness, spacing, and via to reduce reflections and signal attenuation [1]. Dense PCB layouts, curved trace layouts, and asymmetrical traces can cause CM noise and signal integrity issues, resulting in severe electromagnetic interference (EMI) problems [2]. When the return current of high-frequency signals on the microstrip line encounters plane splitting, it is compelled to deviate from the ideal path to return to the source, which generates related CM currents and increases EMI problems [3].

The server system adopts a differential signal structure to increase transmission performance. The differential signal layout requires that the two coupled microstrip lines be parallel and equal in length to each other and that the same differential impedance is maintained between the two lines to avoid EMI problems. To avoid compromising signal integrity, there must be an equal number of vias between the two signal pairs [4]. The common application of differential signals reduces CM noise and provides a continuous current return path using virtual reference plane characteristics [5]. Differential signals have the characteristics of low EMI problems, low crosstalk problems, and strong anti-noise capabilities [6].

Many papers that effectively address the problem related to CM noise using different types of structures such as mushroom-like structures and defected ground structures (DGSs) have been published, and examples of filters described in related papers are described in the following paragraph: A periodically embedded dumbbell-shaped defect structure in DGS structure can maintain a suppression frequency range of 3.3 GHz to 5.7 GHz [7]. Using two U-shaped and one H-shaped coupling patterns, a grounded structure filter can maintain a suppression frequency range of 3.8 GHz to 9.7 GHz [8]. A double-slit complementary split ring resonator filter can maintain a suppression frequency range of 1.52 GHz to 4.07 GHz [9]. A filter that has two enhanced C-shaped couplings can maintain a suppression frequency range of 3.7 GHz to 10.8 GHz [10]. The new filter combines a DGS structure and three load varactor diodes that can maintain a suppression frequency from 2.9 GHz to 8.1 GHz [11]. A double-resonant function filter uses five DGS units that can maintain a suppression frequency range of 2.75 GHz to 4.6 GHz [12]. These DGS structure filters are only capable of suppressing frequencies up to 10.8 GHz and thus cannot support the 32 GHz frequency outlined in server design requirements.

In addition, noise suppression ability can be observed in different mushroom-like structures. For example, a filter using four mushroom structures for negative permittivity metamaterial designs can maintain a suppression frequency range of 3.8 GHz to 7.1 GHz [13]. A filter using a mushroom-like structure and a pair of meandered lines can maintain a suppression frequency range of 1.65 GHz to 5.2 GHz [14]. A quarter-wavelength resonator supports a suppression frequency range of 3.53 GHz to 10.1 GHz [15]. Additionally, a filter that combines multilayer mushroom-like structures and a pair of meandered lines can maintain a suppression frequency range of 3.4 GHz to 13.41 GHz [16]. Filter using a ring structure and serpentine transmission line can maintain a suppression frequency range of 1.57 GHz to 7.87 GHz [17]. These mushroom-like structures are only capable of maintaining suppression frequencies up to 13.41 GHz and thus cannot support the 32 GHz frequency outlined in server design requirements.

A filter using periodically corrugated reference plane (PCRP) structures can maintain a suppression frequency range of 5.3 GHz to 10.4 GHz [18], and filters using defected corrugated reference plane (DCRP) structures can maintain a suppression frequency range of 3.67 GHz to 17.03 GHz [19]. A filter that uses mushroom and DCRP (MDCRP) structures can maintain a suppression frequency range of 5.09 GHz to 20.62 GHz [20]; PCRP, DCRP, and MDCRP structures are only capable of maintaining suppression frequencies up to 20.62 GHz, and thus cannot support the 32 GHz frequency outlined in server design requirements. There are increasing demands for high-speed transmission in the system design of server and computer products. Server manufacturers are also launching related PCIe 5.0 specification server products, and peripheral devices related to PCIe 5.0 specification products have also been proposed. This represents a considerable challenge for system designers because these products need to comply with high-speed transmission design requirements and should be able to solve EMI-related problems caused by high-speed transmission. The goal of this paper is to support a 32 Gb/s PCI Express 5.0 high-speed bus, meeting the requirements of today’s server designs. Therefore, the filter proposed in this work adopts the SMDCRP structure to produce a wider and higher stopband effect. It can reduce EMI problems in servers and computer systems, improving the stability and precision of related controllers and sensors.

## 2. Filter Design and Circuit Modeling

**Figure 2:**
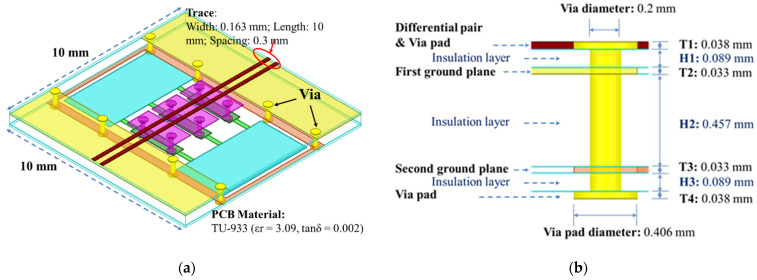
The SMDCRP structure filter: (**a**) the three-dimensional view; (**b**) via-side view [19,20].

The SMDCRP structure filter in this research uses the high-frequency PCB material TU-933, and a three-dimensional view of it is shown in Figure 2a. The filter uses a four-layer PCB. The top layer has two coupled microstrip lines. The first ground plane and the three mushroom structures are in the second layer. There is a second ground plane in the third layer. The fourth layer has only a few via pads. There are eight vias connecting the first and second ground planes. There are six vias connecting the second ground plane to the series-mushroom structure. The PCB has seven layers: three insulation layers and four metal layers. A side view of the via is shown in Figure 2b. All of the via holes go through each PCB layer. The via pad is 0.406 mm, and the diameter of the via hole is 0.2 mm. These structures make up the SMDCRP structure filter.

**Figure 3:**
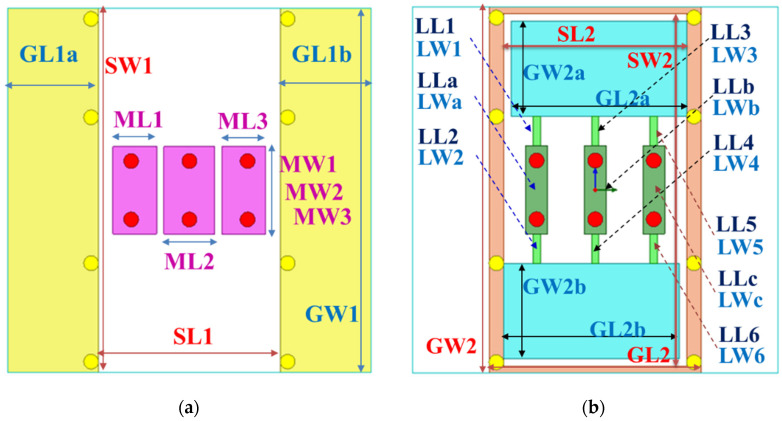
The SMDCRP structure filter: (**a**) the first ground plane; (**b**) the second ground plane.

The first ground plane is split into ground planes GL1a and GL1b; there are three mushroom sheets between the ground planes GL1a and GL1b. Every mushroom sheet uses two vias to connect the second ground plane. The first ground plane is shown in Figure 3a. There are eight vias interconnecting the first and second ground planes. The second ground plane is 5.8 mm × 10 mm and has a slot of 5 mm × 9.8 mm. The second ground plane is shown in Figure 3b. There are corresponding ground sheets and three long ground sheets to connect the second ground plane. These combine into the SMDCRP structure filter, and the relevant dimensions are shown in Table 1.

### 2.1. Design Concept of Filter

**Figure 4:**
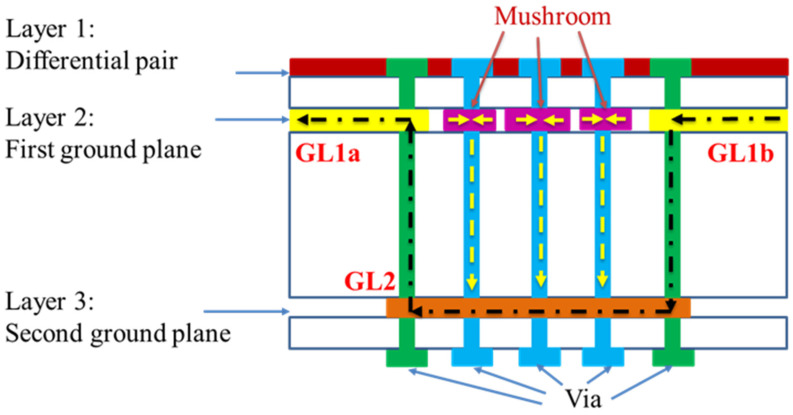
PCB side view of the CM current return path.

Differential signals often have path asymmetry due to very tight PCB layouts, resulting in a large amount of CM noise. Solving the CM noise problem requires a CM noise rejection filter. The proposed design concept utilizes the CM return current path principle in the differential signal and step impedance characteristics changes to achieve noise suppression. Differential mode (DM) signal transmission uses the virtual reference ground, and reference ground damage does not affect differential signal quality. CM signal transmission returns to the source along the reference ground plane, as any damage to the reference ground plane can affect changes in the CM signal. The PCB side view of the CM current return path is shown in Figure 4. The first ground plane is split into ground planes GL1a and GL1b to block the CM current, and the CM current flows to the second ground plane through the vias. The CM current moves smoothly through the three mushroom structures to the second ground plane. These structural changes result in more resonance points and in transmission being close to zero along the CM return current path, resulting in a wider rejection bandwidth. Additionally, the DM signal integrity can be maintained.

We can observe the situation of the CM current return path through the side view of the PCB structure. The CM current flows to the GL1b ground plane and four vias on the right to the second ground plane and then reaches the GL1a plane on the left through the four vias, forming a complete current return path. There are also three CM current return paths, which pass through the three mushroom structures that pass through six vias to the second ground plane, and then reach the GL1a plane on the left through four vias, also forming a complete current return path. Integrating the SMDCRP structure mainly produces more resonance points, making CM noise suppression more broadband and high frequency, and avoiding EMI problems.

### 2.2. Geometric Parameter Design of Filter

This research follows the DCRP and MDCRP structures to develop new filter structures to increase the higher bandwidth of noise suppression capability. After reviewing the relevant DGS and mushroom structure literature, it was found that the mathematical formulas proposed in the literature are difficult to calculate in the SMDCRP structure filter because the SMDCRP structure is complex. Simulation processes were used to understand interrelated changes in geometric parameters.

**Figure 5:**
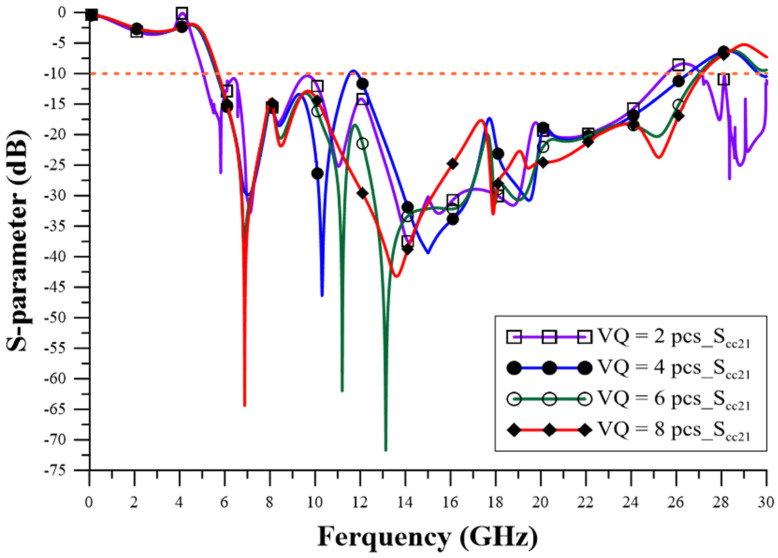
The influence of via quantity (VQ) changes.

**Figure 6:**
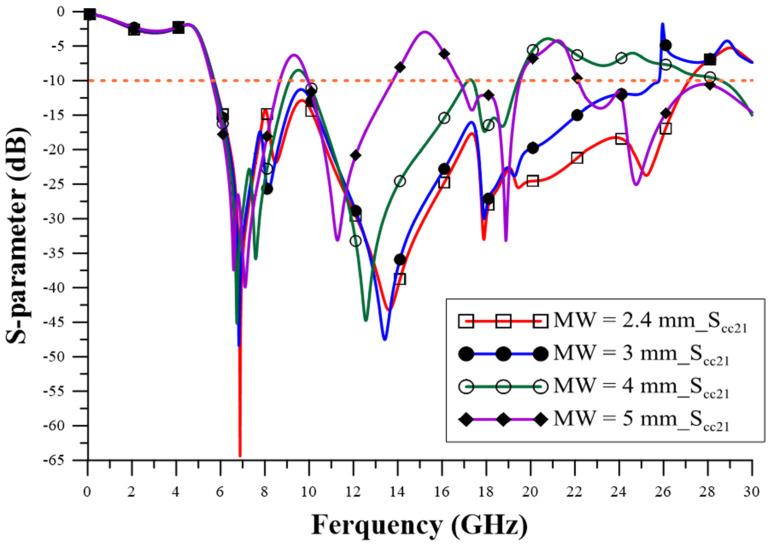
The influence of mushroom width (MW) changes.

**Figure 7:**
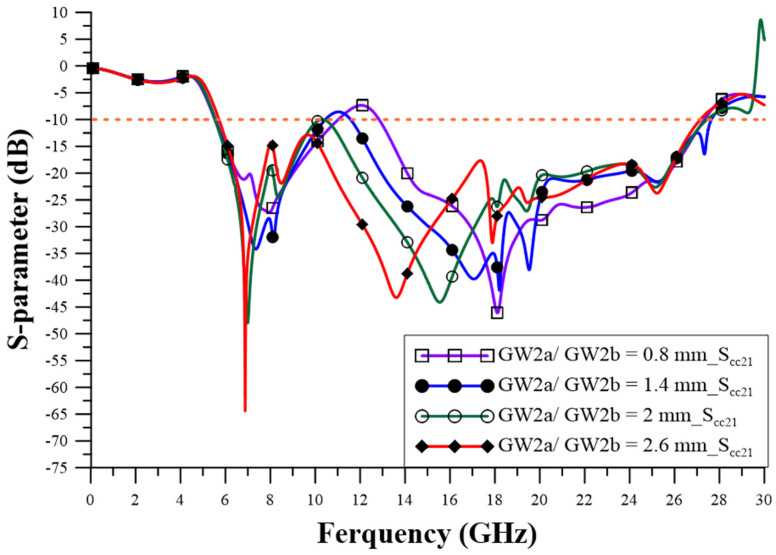
The influence of changes in the width of GW2a and GW2b.

**Figure 8:**
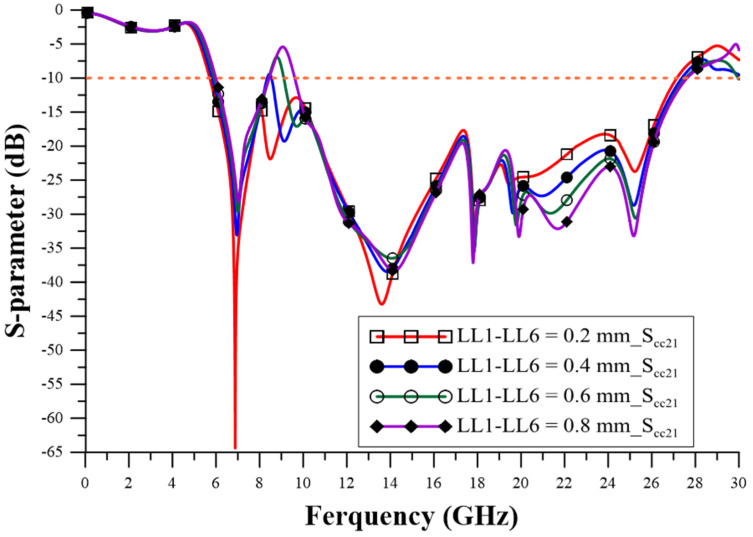
The influence of length changes in LL1–LL6.

**Figure 9:**
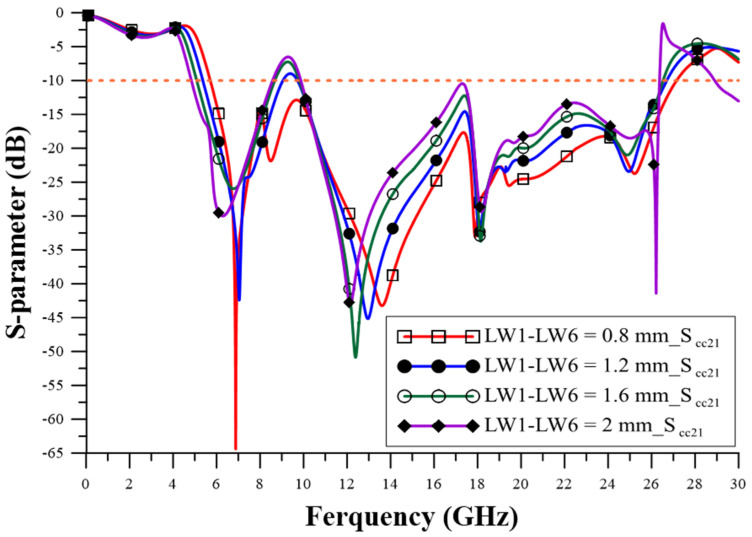
The influence of width changes in LW1–LW6.

The design of the relevant geometric parameters of the filter was carried out using ANSYS HFSS simulation software to achieve the CM suppression effect, which observes the performance of the CM insertion loss (S_cc21_) parameter to determine the best configuration for the geometric parameters. The via quantity (VQ) uses two pieces, four pieces, six pieces, and eight pieces, as shown in Figure 5, and the suppression capability is better when it is increased. The appropriate choice is eight via pieces, as this number can achieve better stopband capability. The series-mushroom width (MW) can be selected as 2.4 mm, 3 mm, 4 mm, and 5 mm, as shown in Figure 6; the stop-band capability is better when it is narrow, so 2.4 mm to was used achieve the width stopband capability. GW2a and GW2b were modified to 0.8 mm, 1.4 mm, 2.0 mm, and 2.6 mm, as shown in Figure 7, and the suppression capability was determined as being better when they were wide, so 2.6 mm is the appropriate size to achieve wider stopband efficacy. LL1–LL6 were changed to 0.2 mm, 0.4 mm, 0.6 mm, and 0.8 mm, as shown in Figure 8. A final size of 0.2 mm was selected to achieve better suppression ability. LW1–LW6 were modified to 0.8 mm, 1.2 mm, 1.6 mm, and 2.0 mm, as shown in Figure 9, and a final size of 0.8 mm was selected to achieve better suppression performance. 

These results prove that the geometric parameter changes in the filter produce more resonance points and that the SMDCRP structure filter achieves a wider suppression bandwidth. Therefore, the results achieved according to the geometric parameter changes are consistent with the theory.

### 2.3. Equivalent Circuit Model

**Figure 10:**
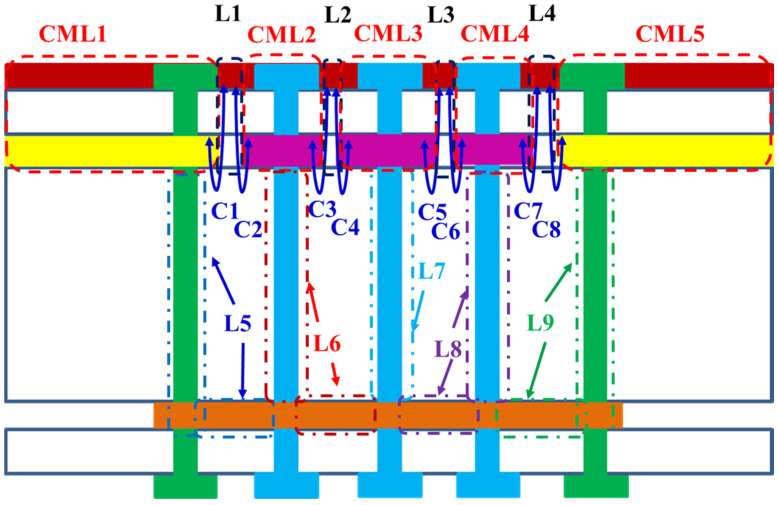
The PCB side view of the SMDCRP structure.

Equivalent circuit modeling can be derived using SMDCRP structure and theory, where DCRP and series-mushroom structures can be observed. The PCB side view of the SMDCRP structure filter is shown in Figure 10. From the PCB structure, it can be observed that the microstrip line is divided into nine parts: five parts form five equivalent coupled microstrip lines CML1, CML2, CML3, CML4, and CML5. The other four parts form four equivalent inductances of L1, L2, L3, and L4, because they do not refer to the ground plane. The two ends of L1, L2, L3, and L4 become the equivalent capacitances of C1, C2, C3, C4, C5, C6, C7, and C8 with the first ground plane and series-mushroom ground planes. The four vias on the left and right connect the second ground plane to form the equivalent inductances of L5 and L9. The six vias in the series-mushroom structure become the equivalent inductances of L6, L7, and L8, connecting the second ground plane. 

**Figure 11:**
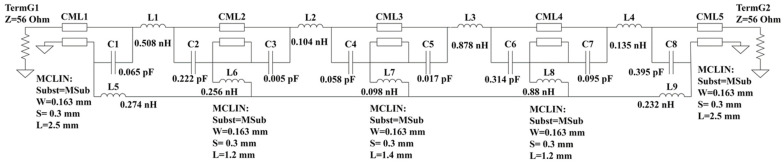
The equivalent half-circuit model of the SMDCRP structure.

The equivalent half-circuit model is constructed in the advanced design system (ADS), and the relevant PCB parameters are imported into the equivalent half-circuit model for simulation in Figure 11. Observing the equivalent half-circuit model produces the CM insertion loss (S_cc21_) parameter. Additionally, the relevant equivalent elements (L1–L9 and C1–C8) are modestly adjusted. Since the realized equivalent half-circuit model is consistent with the simulation results, it can be proven that the equivalent components are valid parameters. The model achieves better noise suppression, modifying the equivalent component values in the equivalent circuit, and changing the CM insertion loss (S_cc21_) parameter is necessary. The low resonance frequency changes when the lower value of L1 results in the low band having poorer CM noise rejection. The low resonance frequency is changed when a higher L2 value results in poorer low-band CM noise rejection. The resonance frequency in the low and high bands changes when there is a lower L3 value, resulting in the entire band having worse CM noise rejection. The resonance frequency in the high band changes when there is a lower L4 value, resulting in worse CM noise rejection in the medium and high bands. The resonance frequency in the entire band changes when there is a lower L5 value, resulting in poor CM noise rejection of the entire band. 

The resonance frequency in the low and medium bands changes when the value of L6 is low, resulting in worse CM noise rejection in the low and medium bands. The resonance frequency in the medium and high bands is changed when there is a lower L7 value, resulting in worse CM noise rejection in the medium and high bands. The low resonance frequency changes when the value of L8 is lower, resulting in worse CM noise rejection in the low band. The high resonance frequency changes when the L9 value is lower, resulting in worse CM noise rejection in the high band.

The low resonance frequency changes when the values of C1 and C6 are high, producing worse CM noise rejection in the low band. The resonance frequency in the low and medium bands changes when the values of C2 and C7 are high, resulting in the poor rejection of CM noise in the low and medium bands. The high resonance frequency changes when the value of C3 is large, resulting in poorer CM noise rejection in the high band. The resonance frequency in the medium band changes when the value of C4 is large, producing poor CM noise suppression in the medium band. The resonance frequency in the low and high bands changes when the values of C5 and C8 are high, and C8 impacts the medium bands, resulting in poor CM noise rejection in the entire band. 

**Figure 12:**
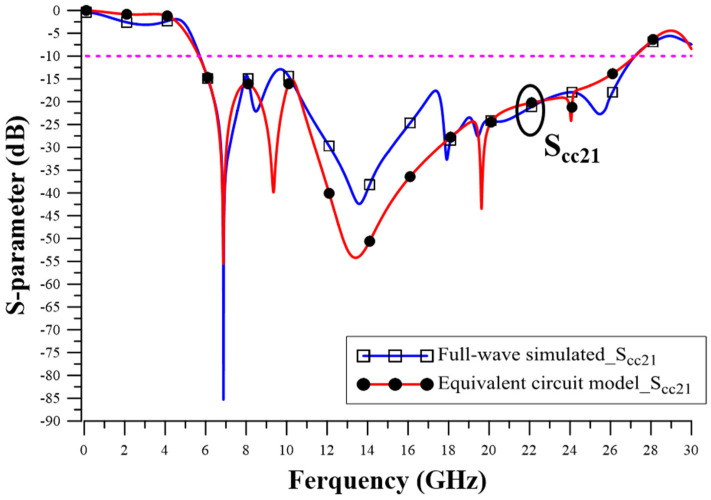
Comparison of equivalent half-circuit model results and full-wave simulated results.

Equivalent half-circuit mode verification is based on full-wave simulation and a comparison between CM insertion loss (S_cc21_) parameters. We can observe that the suppression bandwidth of both is very similar. The insertion loss parameters are also very similar. The comparison between the full-wave simulation results and the equivalent half-circuit model simulation results is shown in Figure 12. The comparison results prove the effectiveness of equivalent circuit modeling.

## 3. Experimental Result

**Figure 13:**
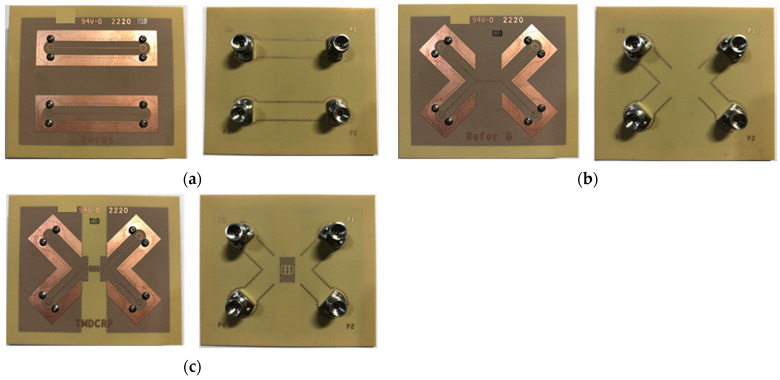
(**a**) The 2× thru the board [19,20]. (**b**) The reference board [19,20]. (**c**) The SMDCRP structure filter board.

The associated mixed-mode scattering parameters were measured using Agilent’s PNA network analyzer N5227A. The entire SMDCRP structure filter used a four-layer PCB. The material used for the PCB was high-frequency TU-933. To make measurement more convenient, the filter size was increased to 60 mm × 50 mm. The CM noise suppression bandwidth performance was verified by actual measurement. The actual measure needs three PCB samples. The 2× thru the board is shown in Figure 13a. To reduce the relative measurement error and realize the real measurements of the device under test, we adopted Keysight’s automatic fixture removal (AFR) calibration technology. The reference board for accelerated comparison is shown in Figure 13b. The verification board for this filter is the SMDCRP structure board shown in Figure 13c.

### 3.1. Frequency Domain Verification

**Figure 14:**
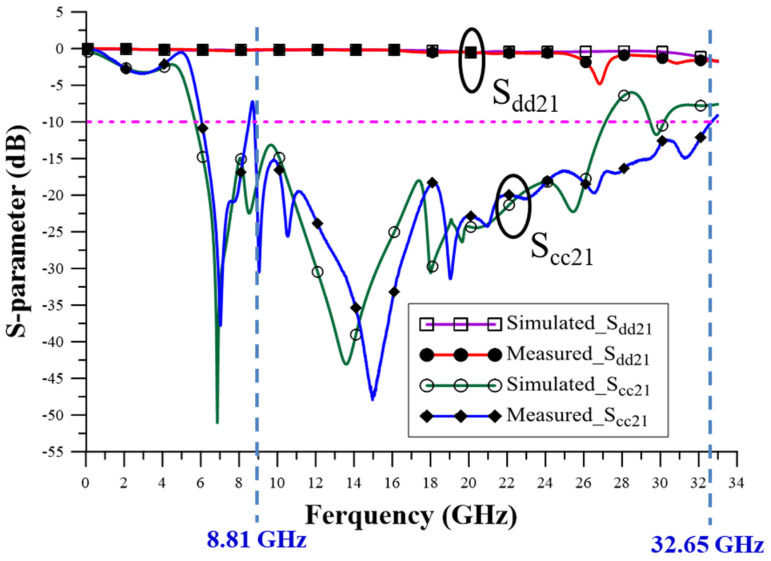
Comparison of simulated and measured results for insertion loss.

Differences can be observed in the simulated and measured insertion loss results. The DM insertion loss (S_dd21_) results are shown in Figure 14. Our actual measurement results were more accurate after the completion of the AFR process, which eliminated related error factors. The simulation results of DM insertion loss in the range from DC to 32 GHz were maintained below −3.162 dB, and the measurement results were below −4.838 dB, proving that adding the SMDCRP structure filter can maintain the signal quality in DM. Additionally, the CM insertion loss (S_cc21_) results can be observed as shown in Figure 14. 

As can be observed by comparing results between the simulation and measurement, the CM noise rejection bandwidth measured is higher than the simulation, which is from 5.72–27.20 GHz to 8.81–32.65 GHz, and the center frequency is measured as higher than the simulated change from 16.46 GHz to 20.73 GHz. The calculated fractional bandwidth of measurement is lower than the simulation frequency band from 130.5% to 115.0%. The poor fractional bandwidth was found to be caused by shifting the center frequency to the high-frequency region due to the measured rejection bandwidth change.

A difference in CM insertion loss (S_cc21_) is observed between the low and high bands in the simulated and measured results. The differences are the result of PCB manufacturing errors. During the manufacturing process, it is difficult to control the consistency of the PCB process and the inductance characteristics of the vias, which results in process-related errors. Here, the changes in the resonant frequency caused the measured results to be inconsistent with the simulated results. The CM noise suppression standard needs to be kept below −10 dB. The measurement results prove that the filter has excellent CM noise suppression ability and high band suppression ability of up to 32 GHz.

### 3.2. Time Domain Verification

The ADS schematic tool can be used to design a simulation circuit to compare the reference board and the SMDCRP structure filter board. Additionally, the measurement results of the two boards generate S4P files, which are input into the ADS schematic tool together with relevant parameters. The internal resistance of the stepping power supply was set to 50 Ω, the input terminal was fed into ±0.5 V, the timing delay was 25 ps, and the rise time was 40 ps. The output results of the analog comparison circuit were fed into Equations (1) and (2), which were calculated as the DM signal output voltage and the CM noise output voltage in the time domain transmission (TDT).

(1) $$V_{TDT-differential}=V_{+}-V_{-}$$

(2) $$V_{TDT-common}=\frac{V_{+}+V_{-}}{2}$$

**Figure 15:**
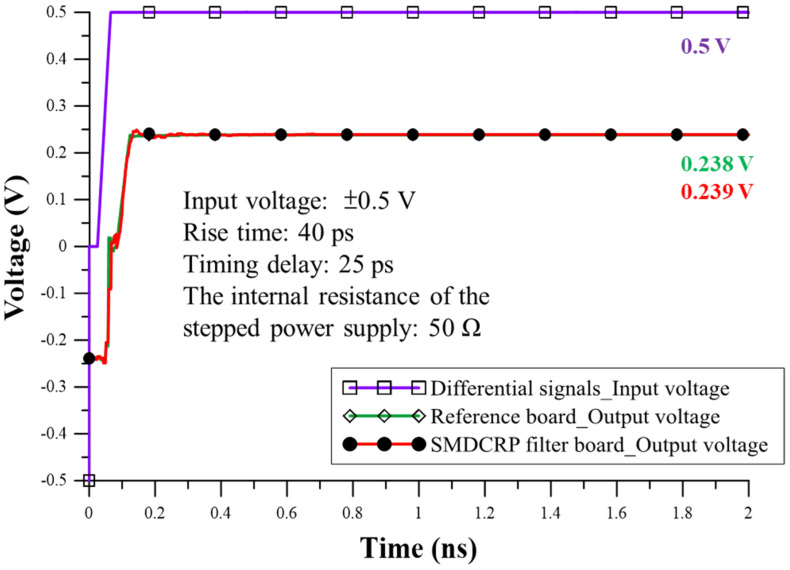
Comparison of DM signal output voltage of the reference and SMDCRP filter boards.

**Figure 16:**
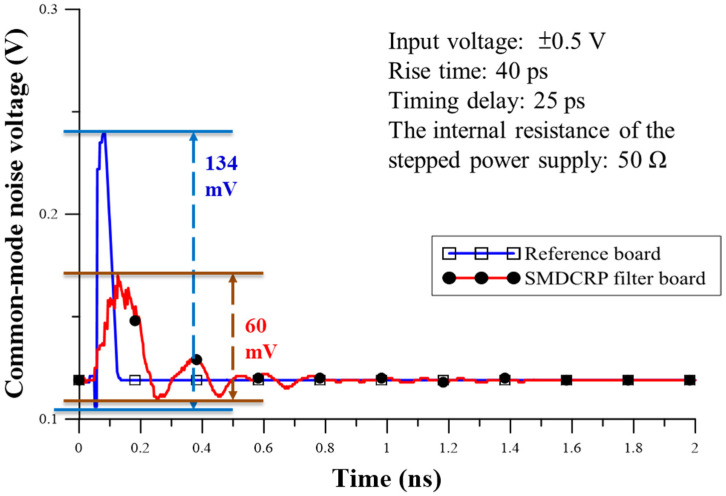
Comparison of CM noise output voltage of the reference and SMDCRP filter boards.

Based on the results obtained using the ADS schematic tool and the relevant calculations, the DM signal output voltage in the TDT is shown in Figure 15. The two board results show that the output DM signal and delay time are similar. The final voltages are 0.238 V and 0.239 V, respectively, indicating that they are very close in terms of voltage. These findings are enough to prove that adding the SMDCRP structure filter does not influence the quality of the DM signal and that it can maintain the integrity of the signal. The CM noise output voltage comparison carried out in TDT is shown in Figure 16 and shows a great contribution to the improvement of CM noise. The measured result is reduced by 60 mV from 134 mV, and the improvement range is about 55.2%. The SMDCRP structured filters are thus demonstrated to have strong CM noise suppression capabilities.

### 3.3. Eye Diagram Verification

Eye diagram measurements were carried out for differential signals of the SMDCRP structure filter board and the reference board and demonstrated the quality of the transmission in the time domain. To create a circuit design to perform eye diagram simulation using the ADS schematic tool, the input terminal voltage was set to ±0.5 V, the rise as was set to 6 ps, the bit rate as set to 32 Gb/s, and the input parameter setting generated a 2^11^−1 pseudorandom binary sequence (PRBS). The S4P files generated by the two boards were imported into the ADS schematic tool for eye diagram simulation. 

**Figure 17:**
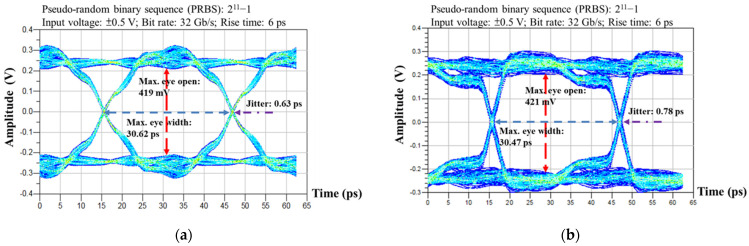
The eye diagram of measured results. (**a**) The reference board. (**b**) The SMDCRP structure filter board.

The differential signal variation between the SMDCRP structure and the reference board can be observed as shown in Figure 17a,b, respectively. Although there are some differences between the two, good eye diagram performance is maintained. These results prove that adding the SMDCRP structure filter to the transmission line of the differential signal does not affect the transmission quality. The relevant eye diagram parameters are provided in Table 2. The maximum eye width, maximum eye open, and jitter of the two structures show some differences when compared numerically, but both hold up well in terms of characteristics. This eye diagram results show that the proposed SMDCRP structured filter can reach the highest transfer rate of 32 Gb/s and support bus function for PCI Express 5.0.

## 4. Discussion

Today’s server and computer systems require buses that support high transmission rates. Different types of structured filters have been described in the literature, such as the DGS, mushroom, PCRP, DCRP, and MDCRP structure filters as shown in Table 3. The DGS, mushroom, and PCRP structure filters can support a highest CM rejection frequency of 13.41 GHz. In addition, the DCRP can support a highest CM rejection frequency of 17.03 GHz. Additionally, MDCRP can support a highest CM rejection frequency of 20.62 GHz. Therefore, it is difficult for filters to use different structures to support high-speed bus requirements according to the system design for current servers.

The SMDCRP structure filter shows the highest suppression frequency in the relevant published literature and supports transmission rates up to 32 Gb/s based on eye diagram results for differential signals. It has good CM noise suppression ability and can maintain signal integrity. The filter supports the high-speed buses required by the system designs for today’s servers. CM noise suppression was determined to be up to 32.65 GHz for the highest frequencies, the center frequency was found to be 20.73 GHz, the fractional bandwidth was calculated as 115.0%, and the filter size was as 1.21 λ_g_ × 1.21 λ_g_. The fractional bandwidth and filter size were calculated based on the center frequency value. Therefore, the SMDCRP structure filter has the highest suppression bandwidth in the relevant published literature. The filter size was calculated to be the largest, and the fractional bandwidth was calculated to be poor.

In research, the geometric parameter simulation process is difficult. It needs to consider all the geometric parameters and correlations and needs to adjust the geometric parameters continuously over a long period to determine the optimal configuration. The research process for equivalent circuits represents another area of difficulty. It is necessary to adjust the relevant equivalent component values over a long period to find out the CM insertion loss parameters results where the equivalent circuit is similar to the simulation. 

In our future work, we will continue to study the absorption of CM filter structures to enable a more optimal design for CM filters as a whole and carry out research to achieve a higher transmission rate of 64 Gb/s to meet the design requirements of next-generation PCI Express 6.0 and new servers. Additionally, we will continue to research the CM filter of multiple parallel transmission channels. Building an EMI-free environment in server systems can improve the accuracy of the associated sensors.

## 5. Conclusions

This paper presented a compact SMDCRP structure for a wideband CM rejection filter. The entire structure used only three PCB layers and can be easily embedded in the PCB design of a server. From the measured results, it has 32 GHz high-frequency CM noise suppression performance and signal integrity can be maintained. According to eye diagram verification, we proved that the transmission rate can be supported by 32 Gb/s, achieving the goal of this study. The SMDCRP structure filters can support the design requirements of SATA Express, HDMI 2.0, USB 3.2, and PCI Express 5.0 in the current servers. Therefore, this system can solve the EMI problem and the stability and accuracy of related controllers and sensors can be improved.

## Figures and Tables

**Table 1:** The geometric parameters of the SMDCRP structure filter.

Parameter	GL1a	GL1b	GW1	SL1	SW1	ML1	ML2	ML3	MW1	MW2
Length (mm)	2.5	2.5	10	5	10	1.2	1.4	1.2	2.4	2.4
**Parameter**	**MW3**	**GL2**	**GW2**	**SL2**	**SW2**	**GL2a**	**SW2**	**GL2a**	**GW2a**	**GL2b**
Length (mm)	2.4	5.8	10	5	9.6	4.8	9.6	4.8	2.6	4.8
**Parameter**	**GW2b**	**LL1**	**LW1**	**LLa**	**LWa**	**LL2**	**LW2**	**LL3**	**LW3**	**LLb**
Length (mm)	2.6	0.2	0.8	0.6	2.4	0.2	0.8	0.2	0.8	0.6
**Parameter**	**LWb**	**LL4**	**LW4**	**LL5**	**LW5**	**LLc**	**LWc**	**LL6**	**LW6**	—
Length (mm)	2.4	0.2	0.8	0.2	0.8	0.6	2.4	0.2	0.8	—

**Table 2:** Comparison of differential eye diagram parameters for measured results.

Parameter	Max. Eye Width	Max. Eye Open	Jitter
Reference Board (32 Gb/s)	30.62 ps	419 mV	0.63 ps
SMDCRP Board (32 Gb/s)	30.47 ps	421 mV	0.78 ps

**Table 3:** The performance comparison of different CM noise suppression filters.

Structure	Rejection Bandwidth	Center Frequency	Fractional Bandwidth	Filter Size	ε_r_	PCB Layers	Year
[7]	3.3–5.7 GHz (−20 dB)	4.5 GHz	53.3%	—	4.4	2	2008
[8]	3.8–9.7 GHz (−10 dB)	6.75 GHz	87.4%	0.44 λ_g_ × 0.44 λ_g_	4.4	2	2009
[9]	1.52–4.07 GHz (−20 dB)	2.795 GHz	91.2%	0.43 λ_g_ × 0.15 λ_g_	3.5	2	2015
[10]	3.7–10.8 GHz (−10 dB)	7.25 GHz	97.9%	0.3 λ_g_ × 0.3 λ_g_	4.4	2	2016
[11]	2.9–8.1 GHz (−15 dB)	5.5 GHz	94.5%	0.36 λ_g_ × 0.24 λ_g_	3.27	2	2019
[12]	2.75–4.6 GHz (−12.25 dB)	3.67 GHz	50.3%	0.28 λ_g_ × 0.18 λ_g_	3.66	2	2020
[13]	3.8–7.1 GHz (−10 dB)	5.45 GHz	60.6%	0.16 λ_g_ × 0.26 λ_g_	—	4	2010
[14]	1.65–5.2 GHz (−10 dB)	3.425 GHz	103.6%	0.11 λ_g_ × 0.11 λ_g_	4.5	4	2012
[15]	3.53–10.1 GHz (−10 dB)	6.815 GHz	96.4%	—	4.4	3	2014
[16]	3.4–13.41 GHz (−10 dB)	8.405 GHz	119.1%	0.25 λ_g_ × 0.25 λ_g_	4.4	5	2017
[17]	1.57–7.87 GHz (−10 dB)	4.72 GHz	133.5%	—	4.4	4	2019
[18]	5.3–10.4 GHz (−10 dB)	7.85 GHz	65.0%	2.74 λ_g_ × 0.55 λ_g_	4.4	3	2016
[19]	3.67–17.03 GHz (−9.29 dB)	10.35 GHz	129.1%	0.60 λ_g_ × 0.60 λ_g_	3.09	3	2023
[20]	5.09–20.62 GHz (−10 dB)	12.85 GHz	120.8%	0.75 λ_g_ × 0.75 λ_g_	3.09	3	2023
Proposed filter	8.81–32.65 GHz (−10 dB)	20.73 GHz	115.0%	1.21 λ_g_ × 1.21 λ_g_	3.09	3	2023

## Data Availability

Not applicable.

## References

[1] Sharawi M.S. (2004). Practical issues in high speed PCB design. IEEE Potentials.

[2] Shiue G.-H., Shiu J.-H., Tsai Y.-C., Hsu C.-M. (2012). Analysis of common-mode noise for weakly coupled differential serpentine delay microstrip line in high-speed digital circuits. IEEE Trans. Electromagn. Compat..

[3] Fornberg P.E., Kanda M., Lasek C., Piket-May M., Hall S.H. (2002). The impact of a nonideal return path on differential signal integrity. IEEE Trans. Electromagn. Compat..

[4] Montrose M.I. (2000). Printed Circuit Board Design Techniques for EMC Compliance—A Handbook for Designers.

[5] Hall S.-H., Heck H.-L. (2009). Advanced Signal Integrity for High-Speed Digital Designs.

[6] Bogatin E. (2010). Signal and Power Integrity—Simplified.

[7] Liu W.-T., Tsai C.-H., Han T.-W., Wu T.-L. (2008). An embedded common-mode suppression filter for GHz differential signals sing periodic defected ground plane. IEEE Microw. Wirel. Compon. Lett..

[8] Wu S.-J., Tsai C.-H., Wu T.-L., Itoh T. (2009). A novel wideband common-mode suppression filter for gigahertz differential signals using coupled patterned ground structure. IEEE Trans. Microw. Theory Techn.

[9] Zhu H.-R., Mao J.-F. (2015). An ultra-wideband common-mode suppression filter based on S-DBCSRR for high-speed differential signals. IEEE Microw. Wirel. Compon. Lett..

[10] Lin D.-B., Lee Y.-H. A wideband common-mode suppression filter using enhanced coupled defected ground structure. Proceedings of the 2016 IEEE International Symposium on Electromagnetic Compatibility (EMC).

[11] Zeng Z., Chen S.J., Fumeaux C. (2019). A reconfigurable filter using defected ground structure for wideband common-mode suppression. IEEE Access.

[12] Savitha R., Karuppiah V. Dual resonant DGS based common-mode filter for high-speed digital circuit applications. Proceedings of the 2020 International Conference on Communication and Signal Processing (ICCSP).

[13] Tsai C.-H., Wu T.-L. (2010). A Broadband and Miniaturized Common-Mode Filter for Gigahertz Differential Signals Based on Negative-Permittivity Metamaterials. IEEE Trans. Microw. Theory Tech..

[14] Hsiao C.-Y., Tsai C.-H., Chiu C.-N., Wu T.-L. (2012). Radiation Suppression for Cable-Attached Packages Utilizing a Compact Embedded Common-Mode Filter. IEEE Trans. Compon. Packag. Manuf. Technol..

[15] Shiue G.-H., Hsu C.-M., Yeh C.-L., Hsu C.-F. (2012). A Comprehensive Investigation of a Common-Mode Filter for Gigahertz Differential Signals Using Quarter-Wavelength Resonators. IEEE Trans. Compon. Packag. Manuf. Technol..

[16] Lin D.-B., Chen Y.-C., Wu C.-T. A broadband filter design for common-mode noise suppression with multilayer mushroom structure in differential transmission line. Proceedings of the 2017 IEEE International Symposium on Electromagnetic Compatibility & Signal/Power Integrity (EMCSI).

[17] Lin D.-B., Chen Y.-H., Zheng Y.-H., Chen B.-Y. A Broadband Common Mode Filter With Embedded Ring Structure. Proceedings of the 2019 Joint International Symposium on Electromagnetic Compatibility, Sapporo and Asia-Pacific International Symposium on Electromagnetic Compatibility (EMC Sapporo/APEMC).

[18] Kim M. (2016). Periodically corrugated reference planes for common-mode noise suppression in high-speed differential signals. IEEE Trans. Electromagn. Compat..

[19] Yu C.-K., Lin D.-B., Lin H.-P. (2023). Wideband common-mode suppression filter using defected corrugated reference plane structures. Microw. Opt. Technol. Lett..

[20] Yu C.-K., Lin D.-B., Lin H.-P. (2023). A Novel Wideband Common-mode Noise Suppression Filter That Combines Mushroom and Defected Corrugated Reference Plane Structures. Appl. Sci..

